# The fallacies of QT correction

**Published:** 2003-10-01

**Authors:** Yash Lokhandwala, SC Toal

**Affiliations:** * Consultant Electrophysiologist PD Hinduja National Hospital, Mumbai; Arrhythmia Associates, Mumbai; Lead Cardiologist, Quintiles ECG Services.; † Fellow Arrhythmia Associates, Mumbai; Medical Director, Quintiles ECG Services.

“Not to correct QT, but how to, that is the question”. The QT interval is a reflection of the action potential in the cardiac cells. Homogenous or heterogenous changes in the action potential duration lead to alteration of QT interval (in addition to morphological changes of T & U waves) [1]. Such changes can be due to change in heart rate & autonomic tone. They can also be markers of abnormal repolarization, depolarization or both as a result of electrolyte disturbances, cardiac diseases, drugs and congenital long QT syndromes [2].

Repolarization disorders are responsible for life threatening arrhythmias like torsades de pointes [2]. The purpose of heart rate correction is to obtain a standardized value that would have been measured in the same subject if the heart rate was 60 beats per minute (QTc). Thus this QTc value will now become independent of the heart rate and measure replarization changes. It will thus be a surrogate marker of the risk of torsade de pointes.

The concept of QTc appeared in 1920, when Bazett introduced his square root formula [3]. This formula obtained from data on 39 young men has been questioned because it overcorrects QT at fast heart rate and undercorrects at low heart rate [4]. Thus at slow heart rate, which is one of the predisposing factors of torsade initiation, Bazett correction can easily mask substantial QT prolongation by under correcting. This can hide the proarrhythmic toxicity of drugs slowing heart rate. An alternative, cube root correction of Fridericia, corrects better than Bazett but again is not reliable at fast heart rates. Compared to these non linear correction formulae, linear regression correction obtained from large population data, like the Framingham heart study linear correction are still better [4].

However the QT does not adapt to changes in heart rate immediately. It takes more than 2 minutes for the QT to adapt (QT/RR hysteresis). Hence correction needs to be done at steady heart rates [1]. The concept of heart rate correction ignores the dynamicity of QT/RR relationship [6]. The QT interval is also under autonomic control. Therefore different modes of heart rate changes, e.g. fast heart rate due to parasympathetic withdrawal versus sympathetic overactivity, lead to different direct and reflex effects on QT prolongation. Hence the standard QTc correction formula will not be representative of the actual repolarization milleu. Ideally each individual should have his own correction derived from multiple ECGs at different heart rates and conditions to get the ideal correction constant. Since this is not feasible a compromise can be made by using formula which have been developed and validated in a large population based cohort like the Framingham study [1].

Alternatively, a table of lower and upper limits of QT interval for different RR cycle lengths can be used by clinicians for references purpose [4]. This method obviates the need of using any QT correction formula. The reliability of this model results from its derivation in a large population based sample. Inspite of all the fallacies of Bazett’s correction, it is still being used clinically. The reason probably lies in its simplicity and the fact that all clinical data signaling risk of torsade are derived from this formula only. But in the near future we feel that the Bazett’s correction will be replaced by a better formula like Fridericia. We also need to be aware that in addition to prolonged QT, an abnormally short QT can also carry dangerous implications of arrhythmogenecity [5]. The QT also represents depolarization events and hence in the presence of depolarization abnormalities like Left Bundle Branch Blocks (LBBB) and Preexcitation syndromes, QTc will not be representative of repolarization abnormalities and should not be commented upon. An alternative interval, the “JT” has been proposed in such cases [6].

In light of all the above fallacies a well designed study looking at different correction formulae and defining one which clinically can risk stratify patients at risk of torsade best, is the need of the hour. There are limitations not only in correcting QT, but also in how it is measured and the reproducibility of these measurements. Therefore, it is possible that in the future the measurement of QT interval may just become an adjunctive, being replaced by more objective and reproducible signs of repolarization abnormalities. Till than ask not why QT, but how to correct QT.
